# How many foods in the UK carry health and nutrition claims, and are they healthier than those that do not?

**DOI:** 10.1017/S1368980015002104

**Published:** 2015-07-09

**Authors:** Asha Kaur, Peter Scarborough, Anne Matthews, Sarah Payne, Anja Mizdrak, Mike Rayner

**Affiliations:** 1 British Heart Foundation Health Promotion Research Group, Centre on Population Approaches for Non-Communicable Disease Prevention, Nuffield Department of Population Health, University of Oxford, Old Road Campus, Oxford OX3 7LF, UK; 2 University of Oxford School of Public Health, Oxford, UK

**Keywords:** Health claims, Nutrition claims, Food labelling

## Abstract

**Objective:**

The present study aimed to measure the prevalence of different types of health and nutrition claims on foods and non-alcoholic beverages in a UK sample and to assess the nutritional quality of such products carrying health or nutrition claims.

**Design:**

A survey of health and nutrition claims on food packaging using a newly defined taxonomy of claims and internationally agreed definitions of claim types.

**Setting:**

A national UK food retailer: Tesco.

**Subjects:**

Three hundred and eighty-two products randomly sampled from those available through the retailer’s website.

**Results:**

Of the products, 32 % (95 % CI 28, 37 %) carried either a health or nutrition claim; 15 % (95 % CI 11, 18 %) of products carried at least one health claim and 29 % (95 % CI 25, 34 %) carried at least one nutrition claim. When adjusted for product category, products carrying health claims tended to be lower in total fat and saturated fat than those that did not, but there was no significant difference in sugar or sodium levels. Products carrying health claims had slightly higher fibre levels than products without. Results were similar for comparisons between products that carry nutrition claims and those that do not.

**Conclusions:**

Health and nutrition claims appear frequently on food and beverage products in the UK. The nutrient profile of products carrying claims is marginally healthier than for similar products without claims, suggesting that claims may have some but limited informational value. The implication of these findings for guiding policy is unclear; future research should investigate the ‘clinical relevance’ of these differences in nutritional quality.

A poor diet is a major modifiable risk factor for many diseases including CVD and cancer^(^ ^1^ ^–^ ^3^ ^)^. Poor diet and low physical activity levels, combined, were estimated to account for 14·3 % of disability-adjusted life years in the UK in 2010, exceeding even the impact of tobacco (11·8 % of disability-adjusted life years)^(^ ^4^ ^)^. Health and nutrition claims could potentially help consumers make healthier food and beverage purchases, and there is some research to show that the presence of health and nutrition claims can influence the perceived healthiness of products^(^ ^5^ ^)^ and can lead to an increase in the sales of products bearing claims^(^ ^6^ ^–^ ^8^ ^)^.

In the UK, food labelling law is determined by the European Union (EU). A health claim is defined by the EU as, ‘any claim which states, suggests or implies that a relationship exists between a food category, a food or one of its constituents and health’^(^ ^9^ ^)^. A nutrition claim is defined as, ‘any claim that states, suggests or implies that a food has particular beneficial nutritional properties due to the energy, nutrients or other substances it contains, contains in reduced or increased proportions or does not contain’^(^ ^9^ ^)^. The EU regulation^(^ ^9^ ^)^ that contains these definitions separates nutrition claims into two sub-categories: (i) ‘nutrient content claims’ that refer to the amount of a nutrient in a product (e.g. ‘low in fat’); and (ii) ‘nutrient comparative claims’ that compare the nutrient levels between two or more products (e.g. ‘lower in fat than …’). An additional sub-category of nutrition claims, i.e. ‘health-related ingredient claims’ that refer to substances other than nutrients or energy, can also be distinguished.

The EU regulation separates health claims into three different sub-categories, here referred to as: (i) ‘nutrient and other function claims’, i.e. health claims that describe the ‘role of a nutrient or other substance in the growth, development and the functions [both physiological and psychological] of the body’ (Article 13 claims); (ii) ‘reduction of disease risk claims’; and (iii) ‘general health claims’. Full definitions for all the different types of health and nutrition claims analysed in the present paper are provided in the online supplementary material. These definitions are those proposed by the International Network for Food and Obesity/non-communicable disease Research, Monitoring and Action Support (INFORMAS), which in turn are based on the definitions for the different types of claim proposed by the Codex Alimentarius Commission (Codex)^(^ ^10^ ^)^. The online supplementary material also shows the equivalent EU definitions where they exist.

While some types of claim e.g. reduction of disease risk or nutrient and other function claims are relatively easy to identify and categorise, some types of health claim are not. For example, claims such as ‘full of goodness’ or ‘to be enjoyed as part of a healthy, active lifestyle’ would be categorised by some as general health claims but not by others. The current study assesses how easily different types of claims can be identified and categorised.

It has been suggested that health and nutrition claims may lead consumers to overestimate the healthiness of products^(^ ^11^ ^)^. In order to reduce this possibility and also the possibility of producers making claims about beneficial aspects of products while ‘hiding’ their non-beneficial properties (e.g. when making a ‘low fat’ claim for a product that is high in salt), the EU regulation proposes that products making health or nutrition claims should meet minimal nutritional criteria. These criteria were to be defined using a nutrient profile model but to date the European Commission has not succeeded in developing such a model. Nutrient profiling has been defined by the WHO as, ‘the science of classifying or ranking foods according to their nutritional composition for reasons related to preventing disease and promoting health’^(^ ^12^ ^)^.

Australia and New Zealand have recently agreed a nutrient profile model, the Nutrient Profiling Scoring Criterion (NPSC), to use for determining the eligibility of products to make a health claim^(^ ^13^ ^)^. The USA has long used a nutrient profile model for such a purpose. In the USA, health claims are permitted only if they do not exceed set thresholds for fat, saturated fat, cholesterol and sodium^(^ ^14^ ^)^.

The proportion of food and beverage products in the UK that carry health and nutrition claims has never been systematically assessed. An audit – simply involving visiting retail outlets and identifying pre-packaged products with health claims – was carried out in the UK in 2003^(^ ^15^ ^)^. The audit examined 372 claims in relation to 182 products and found that most health claims were ‘nutrient and other function claims’. Our study sampled packaged products available through the home-shopping website of the largest retailer in the UK (Tesco) to obtain an accurate estimate of the prevalence of health and nutrition claims within this defined population of foods. To date, it has also not been clear whether those products that carry health or nutrition claims in the UK have a better nutritional profile than those that do not carry them and the current study therefore investigates that question.

The research questions for the present study are therefore:

1. Are health and nutrition claims easily identified and categorised?
2. What is the prevalence of health and nutrition claims for packaged food and non-alcoholic beverage products sold by Tesco in the UK?
3. Do products that carry a health or nutrition claim have a better nutritional profile than products that do not carry such claims?

Questions 2 and 3 are the focus of the current paper.

## Methods

### Sampling

The EU Food Information Regulation defines a ‘pre-packaged foodstuff’ as ‘any single item for presentation as such to the ultimate consumer and to mass caterers, consisting of a foodstuff and the packaging into which it was put before being offered for sale, whether such packaging encloses the foodstuff completely or only partially, but in any case in such a way that the contents cannot be altered without opening or changing the packaging’^(^ ^16^ ^)^. In our study, we were concerned with pre-packaged foods and non-alcoholic beverages that are consumed by adults or children (but not babies, for whom health and nutrition concerns are different). We were not concerned with alcoholic beverages as the claims permitted on these types of products are regulated differently. We included, separately, product items that were available in different sized packages, on the basis that the packaging for the same product in different sized packages may carry different health and nutrition claims.

The products were sampled from the home-shopping website of Tesco, the retailer with the largest market share in the UK at the time (November 2011). The following types of products were excluded prior to sampling:

∙ unpackaged foods;
∙ products that could not be identified as a pre-packaged food or beverage (e.g. there was no description identifying the item as a food, there was no picture, no nutritional information, etc.);
∙ products that were marked as ‘product currently unavailable’;
∙ alcoholic beverages; and
∙ baby and infant foods and beverages.

This left a total of 13 700 packaged products, from which 400 were randomly selected.

The primary research question was what is the prevalence of health and nutrition claims for packaged food and non-alcoholic beverage products sold by Tesco in the UK? A power calculation was conducted to estimate the precision of the prevalence estimates for different numbers of sampled products. After adjustment for a finite population and assuming a prevalence rate for health and nutrition claims of 50 %, 400 products would produce a confidence interval of ±5 %, which was deemed to be precise enough for the purpose of the present study. Three different Tesco branches were visited – two in Oxford and one in Abingdon (Oxfordshire, UK) – between November 2011 and March 2012 in order to purchase the products. The packaging was removed for data extraction. Nutritional information for the content of energy, protein, carbohydrate, sugars, fat, saturated fat, fibre and sodium (g/mg/ml per 100 g/ml) was recorded from the Tesco website, along with information on product name, brand name, manufacturer, package size, price, etc.

### Product categorisation

The products were categorised into fifteen product groups using the FoodEx2 food classification system^(^ ^17^ ^)^. FoodEx2 categorises products into twenty broad product groups, such as grains and grain-based products, vegetable and vegetable products, milk and dairy products and sugar, confectionery and water-based sweets. There are eight levels of sub-categorisation within these broad products groups, each with increasing specificity. The products were also re-categorised at a later stage into five larger product groups for a regression analysis of the nutrient content of products with or without claims. The new categories were: (i) beverages; (ii) fruit, vegetable and grain-based products; (iii) fish, meat and ready meals; (iv) milk and dairy products (including dairy imitates); and (v) miscellaneous products. The first four product groups mapped readily onto FoodEx2 product categories; however, the miscellaneous category contained a variety of products such as confectionery and seasonings which could not be re-categorised into the previous four categories. Further details of the re-categorisation can be found in Table 3 below.

### Claim detection and categorisation

Two researchers applied the INFORMAS taxonomy independently and their decisions were compared (see below). Use of this ‘expert’ taxonomy in the study does not imply that consumers would identify and classify health and nutrition claims in the same way. Little is known about the way consumers would classify claims into health and nutrition claims and their different types if asked to do so.

Health or nutrition claims (as defined above) may take the form of text (e.g. single words, phrases or sentences), brand names (e.g. ‘Healthy Choices’), a symbol, logo or picture (e.g. representing a party of the body or a bodily process), or a prominent web address promising nutritional advice (because we thought that the presence of such a web address implies that the product is generally healthy). In line with the INFORMAS taxonomy the following were not considered to be health or nutrition claims:

∙ the terms ‘natural’, ‘organic’ and ‘Halal’;
∙ information on the absence of additives, preservatives, colourings and flavourings;
∙ allergy advice (e.g. ‘contains nuts’);
∙ statements in relation to specific diets (e.g. dairy and/or lactose free; wheat and/or gluten free; vegetarian or vegan);
∙ storage advice (e.g. ‘stays fresh for longer’);
∙ reference to the presence of a ‘food or food group’ in the product that does not state, suggest or imply a health benefit (e.g. ‘contains chocolate’);
∙ advertising in relation to sport (e.g. ‘official product of the Olympics’) or to health concerns unrelated, or only loosely related, to a healthier diet (e.g. ‘supporting breast cancer research’); and
∙ nutrition labelling, either back-of-pack or front-of-pack (e.g. traffic-light labelling for specific nutrient levels).

Claims were included if they could be observed on any surface of the packaging which is observable to the purchaser. Claims were not included if they could only be observed once the packaging had been opened.

Health and nutrition claims were further categorised according to the nutrient or other food component to which they referred. Nutrient and other function claims were also categorised according to their health-related function or structure as classified by the International Classification of Functioning, Disability and Health (ICF)^(^ ^18^ ^)^ and reduction of disease risk claims were categorised according to the International Classification of Diseases (ICD)^(^ ^19^ ^)^.

### Inter-rater reliability for the detection and categorisation of claims

Inter-rater reliability was assessed for: (i) level of agreement on whether the product packaging contained a health or nutrition claim or not; and (ii) level of agreement on how claims were categorised using the INFORMAS taxonomy. For both of these levels, inter-rater reliability was assessed using kappa scores generated with the statistical software package Stata version 11. All disagreements were then discussed between the study co-authors to reach agreement as to whether the text or graphic should be considered as a health or nutrition claim, and the claim category in which it should be included.

### Comparison of the healthiness of products with or without claims

First, the healthiness of products with or without claims was examined by comparing the difference in mean levels of energy, protein, carbohydrates, sugars, fat, saturated fat, fibre and sodium (per 100 g, generally as sold) between products that carried claims and those that did not, for all products and for different food categories, using *t* tests. The nutrients (plus energy) were selected because their values were available through the Tesco website and are commonly found in nutrient declarations under EU law^(^ ^16^ ^)^.

Differences in nutrient levels shown by these *t* tests could be due to confounding by product group because different product groups were found to have a different prevalence of claims and have different nutritional properties. For this reason a regression analysis was conducted where the results were adjusted for the five broad product groups shown in Table 3. For each nutrient, two regression models were run: (i) model 1 which did not adjust for product group (and therefore produces results that are comparable to the results of the *t* tests); and (ii) model 2 which did adjust for product group.

Second, the healthiness of foods with or without claims was examined using a nutrient profile model: the NPSC. Foods that do not meet the model’s criteria would not be permitted to carry health claims in Australia and New Zealand. The model was applied using Stata version 11 and a logistic regression was conducted to determine the likelihood that a food carrying a claim would meet the model’s criteria.

## Results

### Missing data

Eighteen out the 400 products sampled were unavailable for purchase; therefore 382 products were collected and analysed. Twenty-eight products did not have any nutritional information. These products were included in the claim extraction phase but were not included when assessing the healthiness of products carrying claims. An additional twenty-three products had incomplete nutritional information (one nutrient or more missing) and were included only in the analyses for which data were available.

### Research question 1: are health and nutrition claims easily identified and categorised?

Table 1 shows that there was good agreement between the two researchers over whether or not a food carried a health claim or nutrition claim. Table 1 also shows that for some health and nutrition claims there was more agreement over their type than for others. For example, there was very good agreement over the classification of nutrient comparative claims but less good agreement over the classification of health-related ingredient claims. Nutrient comparative claims and reduction of disease risk claims had the highest percentage agreement (>99 %).

**Table 1:** Inter-rater reliability across the presence and categorisation of claims on a random sample of food and non-alcoholic beverage products available through the website of a national UK food retailer (Tesco), November 2011–March 2012

Agreement type	Agreement (%)	Expected agreement (%)	*κ*	Prob>*Z*
Presence of claims on the 382 products				
Health or nutrition claim	92	59	0·80	<0·001
Nutrition claim	91	59	0·78	<0·001
Health claim	91	75	0·65	<0·001
Claim categorisation of the 274 claims identified by at least one researcher				
Nutrition claim	82	51	0·65	<0·001
Nutrient content claim	89	60	0·77	<0·001
Nutrient comparative claim	99	89	0·87	<0·001
Health-related ingredient claim	85	65	0·58	<0·001
Health claim	84	60	0·60	<0·001
General health claim	86	65	0·60	<0·001
Nutrient or other function claim	97	93	0·66	<0·001
Reduction of disease risk claim	99	97	0·66	<0·001

### Research question 2: what is the prevalence of health and nutrition claims for packaged foods and non-alcoholic beverages sold by Tesco in the UK?

Table 2 shows that nutrition content claims were the most frequent type of claim (sixty-three products with a total of eighty-seven claims), while reduction of disease risks claims were the least common claim type (three products and a total of four claims). Table 2 also shows that in total, 32 % (95 % CI 28, 37 %) of products carried either a health or nutrition claim, with 15 % (95 % CI 11, 18 %) of products carrying at least one health claim and 29 % (95 % CI 25, 34 %) carrying at least one nutrition claim. On the 123 products that carried claims in our sample, we found a total of 263 claims, an average of 2·1 claims per product carrying a claim. Dairy products and beverages were the product categories most likely to carry both health and nutrition claims (Table 3).

**Table 2:** Prevalence of health and nutrition claims on a random sample of food and non-alcoholic beverage products (*n* 382) available through the website of a national UK food retailer (Tesco), November 2011–March 2012

Claim type	No. of products with claims	No. of claims	% of products with claims	95 % CI for % of products with claims
Nutrition claim	111	172	29·1	24·5, 33·6
Nutrient content claim	63	87	16·5	12·8, 20·2
Nutrient comparative claim	17	18	4·5	2·4, 6·5
Health-related ingredient claim	59	68	15·4	11·8, 19·1
Health claim	56	91	14·7	11·1, 18·2
General health claim	46	75	12·0	8·8, 15·3
Nutrient or other function claim	10	12	2·6	1·0, 4·2
Reduction of disease risk claim	3	4	0·8	0·0, 1·7
Health or nutrition claim	123	263	32·2	27·5, 36·9

**Table 3:** Prevalence of claims, by product category, on a random sample of food and non-alcoholic beverage products (*n* 382) available through the website of a national UK food retailer (Tesco), November 2011–March 2012

Description	FoodEx2 categories	No. of products	No. of products with health claims	% of products with health claims	95 % CI for % of products with health claims	No. of products with nutrition claims	% of products with nutrition claims	95 % CI for % of products with nutrition claims	No. of health claims	No. of nutrition claims
Beverages	Coffee, cocoa, tea and infusions Fruit and vegetable juices and nectars Water and water-based beverages	36	10	27·8	13·1, 42·4	19	52·8	36·5, 69·1	16	26
Fruit, vegetable and grain-based products	Fruit and fruit products Vegetables and vegetable products Starchy roots or tubers and products thereof, sugar plants Grains and grain-based product	129	16	12·4	6·7, 18·1	36	27·9	20·2, 35·6	24	57
Fish, meat and ready-meals	Fish, seafood, amphibians, reptiles and invertebrates Meat and meat products Composite dishes	105	10	9·5	3·9, 15·1	17	16·2	9·1, 23·2	17	22
Milk and dairy products (incl. dairy imitates)	Milk and dairy products Products for non-standard diets, food imitates and food supplements or fortifying agents	39	12	30·8	16·3, 45·3	22	56·4	40·8, 72·0	20	38
Miscellaneous foods	Animal and vegetable fats and oils Sugar, confectionery and water-based sweet desserts Seasoning, sauces and condiments	73	8	11·0	3·8, 18·1	17	23·3	13·6, 33·0	14	30
	TOTAL	382	56	14·7	11·1, 18·3	111	29·1	24·6, 33·7	91	173

### Nutrients and ingredients referred to in health and nutrition claims

Table 4 shows that of the 172 different nutrition claims the most common nutrient or ingredient referred to was fat (e.g. ‘less than 2 % fat’) followed by fruit and vegetables (e.g. ‘contains one of your five-a-day’). Nutrition claims that referred to nutrients that, at high levels of intake, can have a damaging effect on health (e.g. fat, sugar or sodium) were more frequent (55 %) than nutrition claims that referred to nutrients with a beneficial impact such as fibre and protein (38 %). Health claims frequently did not refer to a specific nutrient or ingredient. In fact, 70 % of health claims did not do so (e.g. ‘healthy and delicious’).

**Table 4:** Nutrients and ingredients referred to in health and nutrition claims on a random sample of food and non-alcoholic beverage products (*n* 382) available through the website of a national UK food retailer (Tesco), November 2011–March 2012

	Nutrition claims	% of all nutrition claims	Health claims	% of all health claims
Nutrient				
Energy	8	5	7	8
Protein	3	2	2	2
Sugar/sugars	12	7	0	0
Fat	45	26	0	0
Saturated fatty acids	11	6	1	1
Omega 3 fatty acids	2	1	1	1
Fibre	17	10	3	3
Beta-glucan	0	0	1	1
Sodium/salt	6	3	0	0
Cholesterol	1	1	1	1
Folic acid	1	1	0	0
Vitamin C	2	1	0	0
Vitamin D	2	1	0	0
Phosphorus	1	1	0	0
Calcium	7	4	2	2
Magnesium	1	1	0	0
Nitrite	1	1	0	0
Multiple nutrients	11	6	3	3
Ingredient				
Caffeine	3	2	0	0
Fruit and vegetables	23	13	0	0
Wholegrain	7	4	3	3
Other ingredients	4	2	3	3
Unspecified nutrient or ingredient	4	2	64	70
TOTAL	172	100	91	100

### Diseases and health-related functions and structures referred to in health claims

Throughout the entire project, there were four disease risk reduction claims identified. All four of these related to the reduction of risk of CVD (Chapter I51·6 of ICD-10)^(^ ^19^ ^)^. The twelve nutrient and other function claims referred to a broad range of health-related functions and structures (Table 5).

**Table 5:** Health-related functions and structures referred to in nutrient or other function claims on a random sample of food and non-alcoholic beverage products (*n* 382) available through the website of a national UK food retailer (Tesco), November 2011–March 2012

Health-related functions	Health-related structures	Example	No.
Mental functions (B1)	Structures of the nervous system (S1)		0
Sensory functions and pain (B2)	The eye, ear and related structures (S2)		0
Voice and speech functions (B3)	Structures involved in voice and speech (S3)	‘So sip on soya and help build stronger teeth’	3
Functions of the cardiovascular, haematological, immunological and respiratory systems (B4)	Structures of the cardiovascular, immunological and respiratory systems (S4)	‘Oats contain soluble oat fibre which is proven to be good for your heart’	7
Functions of the digestive, metabolic and endocrine systems (B5)	Structures related to the digestive, metabolism and endocrine systems (S5)	‘Dairy free soya drink is naturally kind on tummies’	2
Genitourinary and reproductive functions (B6)	Structures related to genitourinary and reproductive system (S6)		0
Neuromusculoskeletal and movement-related functions (B7)	Structures related to movement (S7)		0
Functions of the skin and related structures (B8)	Skin and related structures (S8)		0

### Research question 3: do products that carry health or nutrition claims have a better nutritional profile than products that do not carry such claims?

Table 6 shows the difference in the amount of selected nutrients between products that carry claims and those that do not using regression analysis. The results of model 1 indicate that, across the board, products with health claims were significantly lower in energy density (232·6 kJ/100 g), fat (6·7 g/100 g), saturated fat (3·1 g/100 g) and sodium (152·8 mg/100 g). When adjusted for product group in model 2, the differences in fat and saturated fat were smaller (5·7 g/100 g and 3·0 g/100 g, respectively) but remained significant. The differences for energy and sodium disappeared and a significant difference in fibre content appeared. A similar pattern of differences was observed for nutrition claims.

**Table 6:** Difference in nutritional quality for products carrying health or nutrition claims compared with those that do not carry health or nutrition claims for a random sample of food and non-alcoholic beverage products (*n* 382) available through the website of a national UK food retailer (Tesco), November 2011–March 2012

	Model 1		Model 2	
Nutrient	Diff.	*P*	Diff.	*P*
Health claims				
Energy (kJ/100 g)	−232·6	0·02	−118·1	0·20
Protein (g/100 g)	+1·1	0·39	+2·1	0·06
Carbohydrates (g/100 g)	+1·5	0·70	+5·3	0·09
Sugars (g/100 g)	−0·6	0·79	−0·4	0·86
Fat (g/100 g)	−6·7	0·00**	−5·7	0·00**
Saturated fat (g/100 g)	−3·1	0·00**	−3·0	0·00**
Fibre (g/100 g)	+0·3	0·44	+0·7	0·05**
Sodium (mg/100 g)	−152·8	0·02**	−97·5	0·14
NPSC odds ratio†	1·61	0·118	1·53	0·182
Nutrition claims				
Energy (kJ/100 g)	−310·6	0·00**	−235·4	0·00**
Protein (g/100 g)	−0·9	0·37	+0·5	0·58
Carbohydrates (g/100 g)	−3·8	0·20	−2·3	0·36
Sugars (g/100 g)	−2·3	0·23	−3·2	0·08
Fat (g/100 g)	−6·1	0·00**	−5·3	0·00**
Saturated fat (g/100 g)	−3·2	0·00**	−3·3	0·00**
Fibre (g/100 g)	+0·2	0·55	+0·5	0·07
Sodium (mg/100 g)	−115·7	0·03**	−56·2	0·30
NPSC odds ratio†	2·18	0·001**	2·22	0·002**

Model 1, no adjustment for food groups; model 2, adjustment for food groups; Diff., difference; NPSC, Nutrient Profiling Scoring Criterion; + denotes an increase; − denotes a reduction. **P<0·05.

† These results were calculated through a logistic regression analysis and report the odds that a product carrying a claim passes the NPSC model before (model 1) and after (model 2) adjusting for food groups.

Products carrying health claims were 61 % more likely to meet the NPSC model criteria than products that did not carry such claims; a difference that was reduced after adjusting for product group (53 %). However these differences were not statistically significant. Products carrying nutrition claims were significantly more (18 %) likely to pass the NPSC model criteria. This difference was increased after adjusting for product group (22 %).

Of the products that did not carry any health or nutrition claims, 51 % (95 % CI 46, 56 %) met the NPSC model criteria, while 62 % (95 % CI 49, 76 %) of the products carrying health claims and 66 % (95 % CI 57, 75 %) of products carrying nutrition claims did so.

Table 7 shows that the main categories responsible for the differences in fat and saturated fat between foods with and without claims were ‘Fish, meat and ready meals’ and ‘Milk and dairy products’. It is products in the ‘Fruit, vegetable and grain-based products’ category which were responsible for the differences in fibre content.

**Table 7:** Difference in nutritional quality for products carrying health or nutrition claims compared with those that do not carry health or nutrition claims, by product category, for a random sample of food and non-alcoholic beverage products (*n* 382) available through the website of a national UK food retailer (Tesco), November 2011–March 2012

	Category									
	Beverages (*n* 36)		Fruit, vegetable and grain-based products (*n* 129)		Fish, meat and ready meals (*n* 105)		Milk and dairy products (including imitates) (*n* 39)		Miscellaneous foods (*n* 73)	
	Diff.	*P*	Diff.	*P*	Diff.	*P*	Diff.	*P*	Diff.	*P*
Health claims										
Energy (kJ/100 g)	+214·7	0·237	+239·2	0·165	−294·9	0·035**	−519·7	0·005	−398·3	0·215
Protein (g/100 g)	+2·0	0·244	+2·8	0·024**	+0·6	0·801	−3·8	0·245	+10·0	0·025**
Carbohydrates (g/100 g)	+7·6	0·284	+16·3	0·010**	−1·9	0·591	−4·5	0·140	+2·7	0·817
Sugars (g/100 g)	+5·4	0·192	+5·6	0·221	−0·3	0·636	−2·7	0·208	−14·3	0·115
Fat (g/100 g)	+1·4	0·258	−2·8	0·332	−7·4	0·030**	−10·2	0·014**	−10·0	0·177
Saturated fat (g/100 g)	+1·3	0·124	−1·2	0·320	−2·4	0·124	−6·3	0·018**	−7·0	0·118
Fibre (g/100 g)	+0·9	0·090	+2·4	0·001**	−0·2	0·582	+0·3	0·112	−1·0	0·481
Sodium (mg/100 g)	+120·9	0·121	−33·8	0·538	−215·0	0·059	−104·3	0·265	−252·8	0·441
Nutrition claims										
Energy (kJ/100 g)	+77·2	0·658	−68·7	0·592	−211·9	0·059	−692·0	0·000**	−402·0	0·092
Protein (g/100 g)	+0·9	0·604	+0·4	0·682	+2·2	0·219	−5·8	0·053	+3·5	0·303
Carbohydrates (g/100 g)	+3·1	0·654	+2·7	0·567	−3·7	0·199	−3·3	0·250	−12·6	0·139
Sugars (g/100 g)	+4·0	0·332	−1·9	0·577	−0·3	0·551	−0·8	0·700	−13·9	0·041**
Fat (g/100 g)	+0·5	0·658	−3·9	0·065	−5·1	0·063	−14·4	0·000**	−4·5	0·414
Saturated fat (g/100 g)	+0·8	0·332	−2·5	0·005**	−2·3	0·065	−9·7	0·000**	−3·4	0·318
Fibre (g/100 g)	+0·8	0·121	+1·5	0·009**	−0·2	0·571	−0·1	0·637	−0·1	0·940
Sodium (mg/100 g)	+92·2	0·232	−5·3	0·900	+20·7	0·818	−169·4	0·054	−222·6	0·367

Diff., difference; + denotes an increase; − denotes a reduction. **P<0·05.

## Discussion

The present study found that health and nutrition claims can be relatively easily identified and categorised. It also found that nutrition claims are almost twice as common as health claims (29 % of products carried at least one nutrition claim compared with 15 % of products carrying at least one health claim). In addition it was found that the nutrient profile of products carrying claims tends to be healthier, in some respects (e.g. in their fat and saturated fat content), than that of products not carrying claims.

We have not yet done a systematic review of previous studies of the prevalence of health and nutrition claims, so here we compare our results with three selected recent studies^(^ ^20^ ^–^ ^22^ ^)^. Our study found a higher prevalence of health claims than the EU-funded project ‘Food labelling to advance better education for life’ (FLABEL)^(^ ^21^ ^)^ but a lower prevalence than the surveys carried out in Australia^(^ ^20^ ^)^ and Ireland^(^ ^22^ ^)^.

The UK arm of the FLABEL study found that health claims and health logos are found on only a small percentage of products (e.g. it found that only 4–6 % of products carried health claims, including symbolic health claims). However, the FLABEL study did not randomly sample across all product categories and instead focused only on five (breakfast cereals, soft drinks, biscuits, yoghurts and pre-packed fresh ready meals). Furthermore, in the FLABEL study the researchers were not required to record the wording of any claims for further analysis.

The Australian survey^(^ ^20^ ^)^ reported a higher prevalence of health and nutrition claims than our study but did not randomly sample across all food categories and instead concentrated on three product groups known to carry a high number of health claims. Similarly a survey of health claims in Ireland^(^ ^22^ ^)^ found that 18 % of products carried a health claim and that 47 % of products carried a nutrition claim; however, the study used a convenience sample that covered only a small number of product categories.

In the present study we found that some types of health and nutrition claims were relatively easy to identify – particularly nutrient content claims, nutrient comparative claims and reduction of disease risk claims. However, health-related ingredient claims and general health claims were more difficult to identify and categorise. These problems have not been identified or quantified in previous studies.

Even using a predefined and agreed approach and a clear taxonomy, there were still disagreements on approximately one in ten products. There were also types of text and graphics that do not seem to have been anticipated by Codex or EU legislators. For example: does a website address that offers healthy eating advice constitute a health claim? We have taken it that it does.

The present study benefited from using a previously developed taxonomy based on international (Codex) definitions. This taxonomy has been developed by the INFORMAS project^(^ ^10^ ^)^. In addition, internationally recognised methods for categorising foods (FoodEx2)^(^ ^17^ ^)^, diseases (ICD-10)^(^ ^19^ ^)^ and health-related functions and structures (ICF)^(^ ^18^ ^)^ were used for the analysis of the results.

Unlike most previous studies of the prevalence of health and/or nutrition claims^(^ ^20^ ^–^ ^22^ ^)^ or food labelling elements in general, the present study used a random sample of products across the majority of food categories, from a defined ‘population’ of foods. While sampling a broad range of products is more costly and time-consuming than sampling specific food categories, it gives a more complete picture of the prevalence of food labelling elements. However, it should be noted that the sampling methods used for our study did not generate a representative sample of all products available for purchase in the UK as products were sampled from just one supermarket (albeit the one with the largest market share). While it has been estimated that up to 90 % of food purchases in the UK are made in supermarkets^(^ ^23^ ^,^ ^24^ ^)^, it might be expected that foods sold in other types of stores would have a higher prevalence of health claims (e.g. health food shops) or a lower prevalence (e.g. discount stores)^(^ ^15^ ^)^.

Another potential weakness of the study is that the nutrient profile model used to assess the healthiness of products with and without claims – the NPSC – was developed for use in Australia and New Zealand and the present study investigated products sold in the UK. However it should be noted that that NPSC model is a modified version of a nutrient profile model first developed in the UK^(^ ^25^ ^,^ ^26^ ^)^.

The statistical power of our study was set to estimate the prevalence of health and nutrition claims in the total population of 13 700 products with an accuracy of ±5 %, but the study is under-powered to estimate differences in prevalence between food categories and under-powered to detect small differences in nutrient content between products that carry and those that do not carry claims.

Our study found what to some might seem to be a surprisingly high prevalence of health and nutrition claims on the packaging of foods and non-alcoholic beverages sold though a major retailer in the UK. This high prevalence, in and of itself, suggests that health and nutrition claims are important ways of marketing such products to consumers. If this marketing is effective, there may be important public health implications to justify the regulation of claims.

The current study also confirms that nutrition claims appear much more frequently than health claims. However, much more attention has been paid to the regulation of health claims – and in particular the comparatively rare forms of health claims, i.e. nutrient and other function claims and disease risk reduction claims – than to the regulation of nutrition claims.

One of the reasons why we identified a high prevalence of health claims was due to our inclusion of general health claims such as ‘healthy’, ‘good for you’, ‘full of goodness’ and ‘consume responsibly as part of a healthy diet’, within the category of health claims. Over 80 % of health claims in our study were classified as general health claims (seventy-five claims on forty-six products). In the future, such claims will only be permitted in the UK if accompanied by a more specific claim. The EU regulation on health and nutrition claims specifies that, from 24 January 2013 (i.e. after the data for the present study were collected), ‘Reference to general, non-specific benefits of the nutrient or food for overall good health or health-related well-being may only be made if accompanied by a specific health claim included in the lists provided for in Article 13 or 14’. It will be interesting to see whether the prevalence of general health claims changes in the light of the implementation of the legislation.

## Conclusions

The present study has also shown that, in general, products that carry health and nutrition claims have a slightly more favourable nutritional profile than those that do not. The main differences were in fat and saturated fat. However, it is difficult to say whether these differences were ‘clinically relevant’. For example, products carrying health and nutrition claims had a fat content that was 6 g per 100 g less than products without claims but it is this enough to justify such claims? At this point we are not clear how best to judge whether our results are ‘clinically relevant’ or not. We think modelling the health effects of consuming products with and without claims could provide some answers but currently we do not have enough data to parameterize a model.

As noted above, the EU regulation^(^ ^9^ ^)^ proposes that there should be a nutrient profile model which sets minimal quality criteria for the nutrition content of products bearing health or nutrition claims. Had the results revealed that products bearing health or nutrition claims were less healthy than products not bearing health or nutrition claims then they would have demonstrated a clear need for further regulation to ensure a minimum nutritional quality for products that carry health or nutrition claims. Our observation that products bearing health or nutrition claims are slightly healthier than foods not bearing claims might imply, on the one hand, that there is no need for such a nutrient profile model or, on the other, that the difference is so slight that a model is urgently required. There is a need for further research that addresses the impact of potential nutrient profile models to regulate health and nutrition claims. Also, more work is required to understand consumer perception and reactions to health and nutrition claims to ascertain whether these claims are informative or misleading.

## Supplementary material

For supplementary material accompanying this paper visit https://doi.org/10.1017/S1368980015002104. click here to view supplementary material
