# Supplementary material for: How many foods in the UK carry health and nutrition claims, and are they healthier than those that do not?
Source: Public Health Nutr. 2015 Jul 9;19(6):988–97. doi: 10.1017/S1368980015002104 (PMC4825057; doi:10.1017/S1368980015002104)
Supplement: Supplementary file 1 [file S1368980015002104sup001.pdf]

Supplementary Materials: Definitions of nutrition claims and health claims (and sub-types) using the INFORMAS taxonomy and EU categories together with examples from foods sold by Tesco

INFORMAS categories	*INFORMAS (Codex) definition*	*EU categories (Regulation 1924/2006 (Amended and corrected) by 04.03.2008*	Examples
Nutrition claim by:	*Nutrition claim* – 'any representation which states, suggests or implies that a food has particular nutritional properties including but not limited to the energy value and to the content of protein, fat and carbohydrates, as well as the content of vitamins and minerals.' (CAC/GL 23-1997)	*Nutrition claim* –' any claim which states, suggests or implies that a food has particular beneficial nutritional properties due to:<br>(a) the energy (calorific value) it<br>(i) provides;<br>(ii) provides at a reduced or increased rate; or<br>(iii) does not provide; and/or<br>(b) the nutrients or other substances it<br>(i) contains;<br>(ii) contains in reduced or increased proportions; or<br>(iii) does not contain;'	
a) Nutrient content claims	*Nutrient content claim* – 'a nutrition claim that describes the level of a nutrient contained in a food [or its energy value]' (CAC/GL 23-1997). [In this taxonomy nutrient content claims include 'Non-addition claims' defined by CAC/GL 23-1997 as ' any claim that a ~~ingredient~~ [nutrient] has not been added to a food, either directly or indirectly. The ~~ingredient~~ [nutrient] is one whose presence or	EU Regulation has no specific name for this type of nutrition claim but gives conditions for this type of claim in Article 8	• low calorie<br>• 90 calories per serving<br>• contains less than 200 calories per serving<br><br>• fat free<br>• virtually fat free<br>• low fat<br>• less than 2% fat<br>• only 2.1% fat<br>• a low fat [blend of filtered water]

INFORMAS categories	*INFORMAS (Codex) definition*	*EU categories (Regulation 1924/2006 (Amended and corrected) by 04.03.2008*	Examples
	addition is permitted in the food and which consumers would normally expect to find in the food']		• low in saturates<br>• [naturally] low in saturated fat<br><br>• high in omega 3<br><br>• free from cholesterol,<br>animal fat<br>[and lactose]<br><br>• no added sugar<br><br>• source of fibre<br>• a good source of fibre<br>• source of [natural] fibres<br><br>• [as] a [natural] source of dietary nitrite [it is attracting much attention from both medical and sports science researchers]<br><br>• [350ml milk] provides 50% of the recommended daily allowance of calcium<br>• includes calcium<br>• with calcium<br><br>• source of calcium and<br>vitamins B12,<br>D2 and

INFORMAS categories	*INFORMAS (Codex) definition*	*EU categories (Regulation 1924/2006 (Amended and corrected) by 04.03.2008*	Examples
			B2<br>• source of vitamin C<br>• now with vit D<br>• 8 vits + Iron<br>• good source of vitamins<br><br>• light thing<br>• light choices (brand)
c) Nutrient comparison claims	*Nutrient comparative claim* – 'a [nutrition] claim that compares the nutrient levels and/or energy value of two or more foods.' (CAC/GL 23-1997)	*Comparative [nutrition] claims*; EU Regulation gives conditions for this type of claim in Article 9	• 33% less fat (than a standard X's vanilla ice cream)<br>• contains 50% less fat* (*than standard X Quiche Lorraine 400g)<br>• reduced fat (30% less than standard X beef)<br><br>• naturally lower in saturates (over 75% lower in saturates than traditional cooking oil)<br><br>• now with 60% less salt (than before Aug 2009)<br>• 90% less salt* (*than our core range of flavours)<br><br>• fewer calories per spoonful (than regular sugar)<br>• lighter
a) Health-related ingredient claims	*Health-related ingredient claim* – any representation which states,	EU Regulation has no specific name for this type of nutrition claim	• 1 of your 5 a day

INFORMAS categories	*INFORMAS (Codex) definition*	*EU categories (Regulation 1924/2006 (Amended and corrected) by 04.03.2008*	Examples
	suggests or implies that a food has particular nutritional properties not related to its energy value or to the content of protein, fat and carbohydrates, vitamins and minerals but related to the content of an ingredient'	but nutrition claims can be for 'substances other than nutrients that have nutritional or physiological effects') (Article 2) and gives conditions for this type of claim in Article 8 and 9.	• contains [healthy] oat [fibre]<br>• 100% plant [goodness]<br>• [tasty white bread] with [the goodness of] wholemeal<br>• […loaves] packed with the wholegrain [goodness needed as part of a healthy diet]<br>• wholegrain guaranteed!<br>• provides one wholegrain serving per bowl [(It is recommended we eat 3 servings of wholegrain a day by USDA)]<br>• low caffeine<br>• Bifidus actiregularis
Health claims by:	*Health claim* – 'any representation that states, suggests, or implies that a relationship exists between a food or a constituent of that food and health.' (CAC/GL 23-1997)	*Health claim* – 'any claim that states, suggests or implies that a relationship exists between a food category, a food or one of its constituents and health;'	
a) General health claims	*General health claim* – a health claim concerning the general beneficial effects of the consumption of foods or their constituents on health.	EU Regulation has no specific name for this type of health claim but acknowledges the existence of this type of claim in Article 10.3	• healthy eating<br>• diet<br>• www.Xdiets.com<br>• X: This system can help you achieve a balanced diet and learn how to

INFORMAS categories	INFORMAS (Codex) definition	EU categories (Regulation 1924/2006 (Amended and corrected) by 04.03.2008	Examples
			make better [great tasting] food choices<br><br>• [X] goodness range<br>• …and not only is it jolly good for you, [but it's really tasty too…]<br><br>• […herbs have been used to] support health [for thousands of years]
b) Nutrient and other function claims	*Nutrient function claim* – 'a [health] ~~nutrition~~ claim that describes the physiological role of the nutrient in growth, development and functions of the body.' (CAC/GL 23-1997) [Although Codex classifies nutrient function claims as nutrition claims it seems more logical to classify them as health claims]<br><br>*Other function claim* – health 'claims concerning specific beneficial effects of the consumption of foods or their constituents, in the context of the total diet on normal functions or biological activities of the body. Such claims relate to a positive contribution to health or to the improvement of a function or to modifying or preserving health.' (CAC/GL 23-1997)	*Health claims describing or referring to the role of a nutrient or other substance in growth, development and the functions of the body; EU* Regulation gives conditions for this type of claim in Article 13 and sub divides such claims into	• includes calcium which helps build stronger teeth and bones<br>• made with calcium and vitamin D which help maintain bones and teeth [(as part of a balanced varied diet with exercise)...]<br><br>• [one 250ml glass of our soya drink] will give you one third of your daily calcium needs, [so sip on soy and help] to build stronger bones and teeth<br>• dairy free soya drink is naturally kind on tummies<br><br>• [oats] contain soluble [oat] fibre which is [proven to be] good for your heart [when eaten regularly]<br>• good for your heart

INFORMAS categories	*INFORMAS (Codex) definition*	*EU categories (Regulation 1924/2006 (Amended and corrected) by 04.03.2008*	Examples
			• fibre helps maintain a [healthy] digestive system<br>• easy to digest<br><br>• consumption of Omega 3 fatty acids [(3g per week), as part of a healthy lifestyle,] helps maintain heart health.<br><br>• [… wholegrain is recommended by nutritionists as it] provides magnesium and phosphorus which are essential for facilitating the body's use of energy and for forming strong bones…
		Health claims describing or referring to psychological and behavioural functions	• calms [and nourishes];<br>• soothes and relaxes;<br>• settles the mind<br>• {enjoy it] and sleep well
		Health claims describing or referring to slimming or weight control or a reduction in the sense of hunger or an increase in the sense of satiety or to the reduction of the available energy from the diet	• Weight watchers (brand name)

INFORMAS categories	*INFORMAS (Codex) definition*	*EU categories (Regulation 1924/2006 (Amended and corrected) by 04.03.2008*	Examples
a) Reduction of disease risk claims	*Reduction of disease risk claim* – health 'claims relating the consumption of a food or food constituent, in the context of the total diet, to the reduced risk of developing a disease or health-related condition.' (CAC/GL 23-1997)	*Reduction of disease risk claim* – 'any health claim that states, suggests or implies that the consumption of a food category, a food or one of its constituents significantly reduces a risk factor in the development of a human disease; EU Regulation gives conditions for this type of claim in Article 14.	• by the time we are 35, 1 in 2 of us has raised cholesterol. Oats contain a soluble fibre called beta glucan which helps soak up cholesterol<br>• provides a third daily requirement of beta-glucan for cholesterol reduction<br>• …can help lower blood cholesterol<br>• the inclusion of at least 25g of soya protein per day [as a part of a diet low in saturated fat] can help reduce cholesterol in the blood.<br><br>• Extra sugarfree gum is beneficial for dental health as it helps to neutralise plaque acids
No specific category in INFORMAS. Claims classified either as nutrient and other function claims, general health claims or reduction of disease risk claims		*Health claims referring to children's development and health;* EU Regulation gives conditions for this type of claim in Article 14	
